# Thermal challenge of the microcirculation in patients after cardiac surgery

**DOI:** 10.1186/2197-425X-3-S1-A416

**Published:** 2015-10-01

**Authors:** D Orbegozo Cortes, G Stringari, R Damazio, D De Backer, J-L Vincent, J Creteur

**Affiliations:** Hopital Erasme - Intensive Care, Universite Libre de Bruxelles, Brussels, Belgium; Universite Libre de Bruxelles, Brussels, Belgium

## Introduction

Microcirculatory alterations have been correlated with poorer outcomes in different populations of critically ill patients. A controlled increase in the local skin temperature (thermal challenge) induces vasodilation, which may represent a measure of microcirculation recruitability and can be easily evaluated non-invasively using skin laser Doppler (SLD) (1). We previously demonstrated lower SLD values during thermal challenge in patients with circulatory shock, particularly in non-survivors (2).

## Objectives

The aim of this study was to evaluate whether reduced microcirculatory recruitability could be identified using this technique in patients after cardiac surgery and, if so, whether it was correlated with outcome.

## Methods

We evaluated 28 patients admitted to our department of intensive care after cardiac surgery. A thermostatic SLD probe (Perimed, Sweden) was placed on the proximal anterior forearm to evaluate the skin blood flow (SBF), measured in perfusion units (PU). The probe temperature was set at 37°C and we recorded the basal SBF. The temperature was then increased to 43°C and the SBF evaluated 9 min later. We calculated the ratio between the two values as an index of capillary recruitment. We compared patients who died or stayed in the ICU for more than 72 hours (COMPLICATED) with those who survived and stayed less than 72 hours in the ICU (CONTROL). All values are presented as median values and interquartile range. We calculated the area under the receiver operating characteristics curve (AUC-ROC) for the SLD ratio 43/37°C to discriminate between the two groups (COMPLICATED vs CONTROL). All analyses were performed using SPSS 22.0 (IBM, USA).

## Results

The main patient characteristics are shown in Table 1. SBF was similar between groups at 37°C, but lower at 43°C in the COMPLICATED than in the CONTROL group (Table 2). The AUC-ROC (CI 95%) for the SLD ratio was 0.76 (0.58-0.94).

**Table 1:** Main characteristics of both groups.

VARIABLE	CONTROL (n = 16)	COMPLICATED (n = 12)	P
**Age (years)**	67 ± 13	69 ± 9	0.65
**Coronary disease n(%)**	8(50)	8(67)	0.68
**Pre-surgery left ventricular ejection fraction (%)**	60(38-60)	57(28-60)	0.54
**Cardiopulmonary bypass time (min)**	122(94-130)	127(121-148)	0.31
**Hemoglobin (g/dL)**	10.9(10.0-11.5)	9.7(9.3-10.4)	0.09
**APACHE II score**	15 ± 4	21 ± 6	< 0.01
**ICU length of stay (days)**	1.0(0.9-1.9)	6.8(4.2-11.5)	< 0.01
**Mortality n(%)**	0(0)	3(25)	0.07

**Table 2:** *Hemodynamic and SLD variables.*

VARIABLE	CONTROL (n = 16)	COMPLICATED (n = 12)	P
**Cardiac index (L/min/m²)**	2.5(2.0-2.8) (n = 7)	1.9(1.8-2.4) (n = 12)	0.24
**Mean arterial pressure (mmHg)**	79 ± 8	72 ± 9	0.04
**Central venous pressure (mmHg)**	10 (6-12)	11 (9-12)	0.83
**Lactate (mEq/L)**	1.4 (1.1-2.3)	2.1 (1.6-3.0)	0.13
**Norepinephrine (mcg/Kg/min)**	0.0(0.0-0.1)	0.2(0.0-0.3)	0.01
**Time from ICU admission to SLD test (hours)**	2(1-6)	2(1-15)	0.43
**Skin blood flow 37°C (PU)**	12.8(10.1-21.7)	13.0(9.3-19.6)	0.71
**Skin blood flow 43°C (PU)**	81.8(47.1-126.3)	38.9(18.0-66.6)	0.03
**Skin blood flow ratio**	5.8(3.2-8.0)	2.9(1.8-3.8)	0.02

## Conclusions

Patients with a complicated course after cardiac surgery have reduced microcirculatory recruitability as assessed by a non-invasive SLD thermal challenge in the first hours during the ICU stay.

## Grant Acknowledgment

Institutional funds
